# Comprehensive response criteria for myeloid/lymphoid neoplasms with eosinophilia and tyrosine kinase gene fusions: a proposal from the MLN International Working Group

**DOI:** 10.1038/s41375-023-01859-3

**Published:** 2023-04-19

**Authors:** William Shomali, Philomena Colucci, Tracy I. George, Jean-Jacques Kiladjian, Cheryl Langford, Jay L. Patel, Andreas Reiter, Alessandro M. Vannucchi, Jason Gotlib

**Affiliations:** 1 Division of Hematology, Stanford Cancer Institute/Stanford University School of Medicine, Stanford, CA USA; 2 Incyte Corporation, Wilmington, DE USA; 3 ARUP Laboratories and University of Utah School of Medicine, Salt Lake City, UT USA; 4 Hôpital Saint-Louis, Paris, France; 5 Universitätsmedizin Mannheim, Mannheim, Germany; 6 University of Florence, Azienda Ospedaliero-Universitaria Careggi, Florence, Italy

**Keywords:** Myeloproliferative disease, Myeloproliferative disease

## Introduction

In 2008, in response to the identification of patients with “chronic eosinophilic leukemia” or “hypereosinophilic syndrome” who carried recurrent tyrosine kinase fusion genes involving *PDGFRA*, *PDGFRB*, or *FGFR1*, the World Health Organization classification of myeloid neoplasms included a new category termed “Myeloid/lymphoid neoplasms (MLN) with eosinophilia and rearrangements of *PDGFRA, PDGFRB*, or *FGFR1*” [1]. This World Health Organization category was revised in 2016 with the addition of *PCM1::JAK2* as a provisional entity [1]. In the recent fifth edition of the World Health Organization classification, similar to the recent update to the International Consensus Classification, the category was renamed to “myeloid/lymphoid neoplasms with eosinophilia and tyrosine kinase gene fusions” and both classifications added novel subtypes with new *JAK2* rearrangements (e.g., *BCR::JAK2*, *ETV6::JAK2*) as well as fusions involving *FLT3*, and the *ETV6*::*ABL1* fusion [2, 3]. Although eosinophilia (>0.5 × 10^9^/l) or hypereosinophilia (>1.5 × 10^9^/l) are characteristic of this subgroup, they are not universally present [4]. The clinical phenotype is largely influenced by the involved tyrosine kinase fusion gene and/or the fusion partner gene [5, 6]. For example, most patients with the *FIP1L1::PDGFRA* fusion gene present with a chronic myeloid neoplasm with eosinophilia; however, mixed lineage presentations are more common in patients with *FGFR1* fusions [5–8]. Furthermore, in MLN with *FGFR1* rearrangements, translocations involving *ZMYM2* are more commonly associated with a T-lymphoblastic lymphoma phenotype, whereas translocations involving *BCR* tend to lead to a phenotype resembling *BCR::ABL1* positive chronic myeloid leukemia [5, 6, 9].

Treatment of patients with MLN and rearrangements of *PDGFRA*, *PDGFRB*, or *FGFR1* is dependent upon the involved tyrosine kinase fusion gene [5, 10, 11]. Imatinib is associated with complete hematologic, cytogenetic, and molecular responses in patients with *PDGFRA*- and *PDGFRB*-rearranged MLNs and is approved by the US Food and Drug Administration for these indications [4, 10–12]. Pemigatinib was approved for relapsed/refractory MLN with *FGFR1* rearrangement in August 2022 [13].

Here we propose comprehensive response criteria based on the heterogenous clinical presentations of patients with MLN with eosinophilia and tyrosine kinase gene fusions. The MLN International Working Group (MLN IWG) was formed to adjudicate diagnoses and treatment responses in the FIGHT-203 study of pemigatinib in MLN with *FGFR1* rearrangements.

## MLN with *FGFR1* rearrangements

MLN with *FGFR1* rearrangement was previously known as 8p11 myeloproliferative syndrome [14]. The defining cytogenetic abnormality, a translocation at the 8p11 locus, was found to involve the *FGFR1* gene [14]. Table 1 lists the diagnostic criteria for MLN with *FGFR1* rearrangement. Clinical presentation can be in the form of chronic phase (CP) of a myeloid neoplasm detected in the bone marrow (BM)/peripheral blood (PB) (e.g., myeloproliferative neoplasm [MPN], myelodysplastic syndrome [MDS], or MDS/MPN), or blast-phase (BP) disease detected in the BM/PB (e.g., acute myeloid leukemia [AML], T- or B-cell acute lymphoblastic leukemia [ALL], mixed phenotype acute leukemia [MPAL]), and/or extramedullary disease (EMD) that is recognized as a BP component [4, 5, 7, 8]. Different phases and lineages of the disease can be seen in the same patient (e.g., chronic myeloid neoplasm in the bone marrow with concomitant T-cell acute lymphoblastic lymphoma in an EMD site) [4, 5]. Further, both primary BP disease and secondary BP disease as a consequence of rapid progression from CP, usually within 1–2 years, are reported in many patients [6].

**Table 1:** Diagnostic criteria for MLN with *FGFR1* rearrangement [1–3].

Bone marrow involvement with a chronic myeloid neoplasm, usually an MPN or MDS/MPN invariably with eosinophilia, neutrophilia, or monocytosis OR Bone marrow involvement with blast-phase disease, either B- or T-ALL, AML, or MPAL sometimes with bone marrow or peripheral eosinophilia
AND/OR
Extramedullary involvement with a blast-phase malignancy, either B- or T-ALL, AML, or MPAL
AND
Presence of t(8;13) (p11;q12) or variant 8p11 translocation leading to *FGFR1* rearrangement in myeloid cells, lymphoblasts, or both

*ALL*, acute lymphoblastic leukemia; *AML*, acute myeloid leukemia; *MDS*, myelodysplastic syndrome; *MPAL*, mixed phenotype acute leukemia; *MPN*, myeloproliferative neoplasm.

**Fig. 1:**
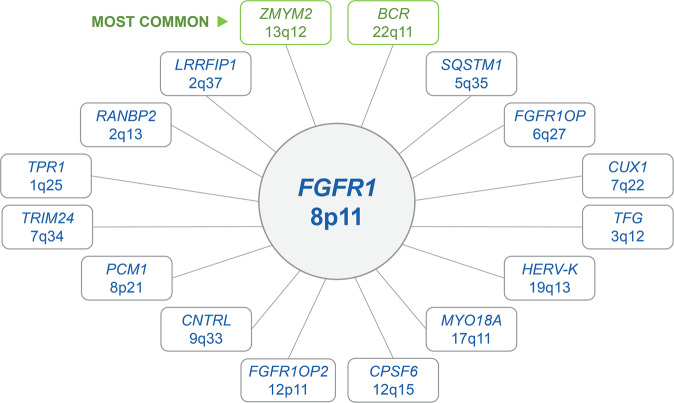
*FGFR1* fusion partners. Sixteen fusion partners of *FGFR1* have been currently characterized. Chromosome breakpoints for the fusion partners are shown below each partner gene. *ZMYM2* on chromosome 13q12 and *BCR* on chromosome 22q11 are the most common fusion partner genes of *FGFR1*.

Sixteen fusion partners to *FGFR1* have been reported, with t(8;13)(p11;q12) involving *ZMYM2* being the most common [6, 15, 16]. *FGFR1*-associated translocations or alterations can be identified with conventional cytogenetic analysis and confirmed with fluorescence in situ hybridization (FISH) using *FGFR1* break-apart probes [5, 11]. Infrequently, *FGFR1* rearrangements are cryptic by conventional cytogenetic analysis and can only be detected by FISH, reverse transcriptase-polymerase chain reaction (RT-PCR), and/or next-generation sequencing analysis [5, 17]. Fig. 1 summarizes the known gene fusion partners to *FGFR1* and their respective translocations.

Treatment with multikinase inhibitors with nonspecific anti-FGFR1 activity, including ponatinib and midostaurin, only provide short-term hematologic responses and rarely result in cytogenetic responses [6, 8, 18, 19]. Current treatment of patients with CP disease includes hydroxyurea or one of the multikinase inhibitors with nonspecific anti-FGFR1 activity [7, 8]. Treatment of patients with BP disease includes intensive induction chemotherapy followed by allogeneic hematopoietic stem cell transplantation in patients achieving disease control [5, 7, 8]. However, the option of allogeneic hematopoietic stem cell transplantation may be limited by patient age and comorbidities and lack of response to chemotherapy. In a review of 45 patients, 14 with CP and 31 with BP, the 1-year overall survival was 43.1%, and 46.2% of patients with CP disease progressed to BP at 1 year [7].

## Fight-203 study

FIGHT-203 is a phase 2, open-label, multicenter study evaluating the efficacy and safety of pemigatinib (INCB054828) in adult patients with MLN with *FGFR1* rearrangements [20]. Pemigatinib is a selective and potent inhibitor of FGFR 1–3 and is approved for the treatment of patients with advanced or metastatic cholangiocarcinoma with *FGFR2* rearrangements [21, 22].

Patients enrolled in FIGHT-203 have a documented MLN with an 8p11 translocation on standard karyotyping and/or evidence of an *FGFR1* rearrangement on break-apart FISH. Most patients enrolled in the study had at least one prior therapy; however, treatment-naive patients were also enrolled [20]. The primary endpoint of FIGHT-203 is complete clinical response (CR) rate. Secondary endpoints include overall response rate defined as the percentage of patients who achieved a best overall response of CR or partial response (PR), and cytogenetic response rates based on conventional cytogenetics or break-apart FISH (complete cytogenetic response and partial cytogenetic response) [20]. Primary and secondary endpoints were assessed by the investigators according to protocol-defined criteria [20]. In addition, a Central Review Committee (CRC), also known as the MLN IWG, consisting of hematopathologists and hematologists, convened regularly to retrospectively review and adjudicate diagnoses and responses. The CRC developed comprehensive response criteria based on the heterogeneous clinical presentations of patients enrolled in the trial. The criteria are a composite of previously published response criteria for MDS/MPN, acute leukemia, and lymphoma [23–25]. During their review, the CRC members discussed the histopathologic, laboratory, and radiologic results, and arrived at a consensus decision to assign patients to respective categories of clinical presentation. For the adjudication of each clinical and cytogenetic response, committee members reviewed and discussed the data, and assigned a response by consensus.

## Categories of clinical presentation

During the CRC adjudication, it became evident that response criteria were needed that could address both the CP and BP presentations, as well as the potential presence of EMD.

The following five potential clinical presentation categories may be seen in MLN with *FGFR1* rearrangement that are also applicable to other MLNs with tyrosine kinase fusion genes: (1) CP disease involving the BM/PB without EMD; (2) CP disease involving the BM/PB with concurrent EMD; (3) BP disease involving the BM/PB without EMD; (4) BP disease involving the BM/PB with EMD; and (5) EMD only (ie, without evidence of BM/PB involvement). In addition to these five presentation categories, and because the majority of patients had received therapy before enrollment in the study, the CRC recognized a sixth category to reflect those patients with evidence of persistent 8p11 cytogenetic abnormality/*FGFR1* rearrangement but without morphologic and/or radiologic evidence of disease in the BM/PB or EMD.

## Response criteria based on clinical presentation

### Criteria for chronic-phase disease in the bone marrow and peripheral blood

The CP disease response criteria adopted by the CRC (Table 2) are modified from the IWG for MDS/MPN proposed criteria and require evaluation of the spleen/liver by palpation, BM, PB smear, and complete blood count with differential [23]. Criteria for CR, complete response with partial hematologic recovery (CR_h_), PR, stable disease, loss of response, and progressive disease (PD) are summarized in Table 2.

**Table 2:** Response criteria for chronic-phase disease in the bone marrow/peripheral blood.

Response	Criteria
**CR**	**Bone marrow:** • ≤5% blasts • Normal maturation of all cell lines (no or minimal dysplasia) • Normal age-adjusted cellularity^a^ • Bone marrow fibrosis grade 0–1^b^ **Peripheral blood:** • WBC ≤10.0 × 10^9^/L • Hb ≥11 g/dL • Platelets ≥100 × 10^9^/L and ≤450 × 10^9^/L • Neutrophils ≥1 × 10^9^/L • Monocytes ≤1 × 10^9^/L • Eosinophils ≤0.5 × 10^9^/L • Blasts = 0% • Neutrophil precursors ≤2% **Hepatosplenomegaly** • Resolution of hepatosplenomegaly by palpation **Comments:** • CR may still be assigned if bone marrow cellularity and fibrosis are not available/evaluable • CHR can be assigned if all peripheral blood criteria are met • If blasts and neutrophil precursors were not present at baseline, CR and CHR may still be assigned if peripheral blood smear is not available/evaluable
**CR**_**h**_	**Bone marrow:** • ≤5% blasts • Normal maturation of all cell lines (no or minimal dysplasia) • Bone marrow fibrosis grade 0–1^b^ **Peripheral blood:** • WBC ≤10.0 × 10^9^/L • Hb ≥8 g/dL • Platelets ≥50 × 10^9^/L and ≤450 × 10^9^/L • Neutrophils ≥0.5 × 10^9^/L • Blasts = 0% • Monocytes ≤1 × 10^9^/L • Eosinophils ≤0.5 × 10^9^/L **Hepatosplenomegaly** • Resolution of hepatosplenomegaly by palpation **Comments:** • CR_h_ may still be assigned if bone marrow fibrosis is not available/evaluable • If blasts were not present at baseline, CR_h_ may still be assigned if peripheral blood smear is not available/evaluable
**PR**	**Bone marrow:** • Reduction of blasts by ≥50% (remaining ≥5%) **Peripheral blood:** • WBC ≤10 × 10^9^/L • Hb ≥8 g/dL • Platelets ≥50 × 10^9^/L and ≤450 × 10^9^/L • Neutrophils ≥0.5 × 10^9^/L • Reduction of blasts by ≥50% if present at baseline • Reduction of AMC and/or AEC by ≥50% if increased at baseline (remaining AMC >1 × 10^9^/L and/or AEC >0.5 × 10^9^/L)
**SD**	Absence of CR, CR_h_, PR, and criteria for PD not met
**LOR**	Increase in bone marrow and/or peripheral blood blasts by ≥50% after initially achieving a CR, CR_h_, or PR
**PD**	Increase in bone marrow and/or peripheral blood blasts by ≥50% without first achieving a CR, CR_h_, or PR
**NE**	Tissue not available and/or evaluable

^a^Age-related normal % cellularity: adults 30–70 years → 40–70%. Adults >70 years → ≤25% [28].

^b^European Consensus Criteria for Fibrosis are used for this grading, MF-0 to MF-3 [29].

*AEC* absolute eosinophil count, *AMC* absolute monocyte count, *CHR* complete hematologic response, *CR* complete response, *CR*_*h*_ complete response with partial hematologic recovery, *Hb* hemoglobin, *LOR* loss of response, *NE* not evaluable, *PD* progressive disease, *PR* partial response, *SD* stable disease, *WBC* white blood cells.

A CR_h_ category was included in the response criteria. CR_h_ meets the criteria for CR except there is no requirement for normal age-adjusted cellularity and allows cytopenias defined as hemoglobin ≥8 g/dL, platelet count ≥50 × 10^9^/L, and absolute neutrophil count ≥0.5 × 10^9^/L. Historically, this category of “less than CR” was first introduced in AML response criteria with the progressive use of less intensive therapies in the treatment landscape [26], where patients receive treatment up to the day of response assessment. Consequently, myelosuppressive effects of therapy may confound response assessment by preventing full recovery of blood counts in the absence of morphologic evidence of AML [26]. Similarly, worsening cytopenias were observed with the use of KIT inhibitors in advanced systemic mastocytosis, and the CR_h_ category was introduced to the modified IWG response criteria used in the evaluation of avapritinib in advanced systemic mastocytosis [27]. In the context of advanced systemic mastocytosis, the CR_h_ category recognizes that in the absence of evidence of systemic mastocytosis due to successful treatment, persistently low blood counts may instead relate to treatment-associated myelosuppression or the presence of a concomitant-associated hematologic neoplasm.

### Criteria for blast-phase disease in the bone marrow and peripheral blood

The response criteria for BP disease in the BM were largely based on the published response criteria for acute leukemia as summarized in Table 3 [25]. PR was modified to include partial hematologic recovery consisting of (1) an absolute neutrophil count >0.5 × 10^9^/L and (2) platelet count >50 × 10^9^/L.

**Table 3:** Response criteria for blast-phase disease in the bone marrow/peripheral blood.

Response Category	ANC, × 10^9^/L	Platelets, 10^9^/L	Bone Marrow Blasts, %	Peripheral Blood Blasts, %	Comments
CR	>1	>100	<5	ND	Complete hematologic response should be noted if all peripheral blood criteria met
CR_i_^a^	<1	<100	<5	ND	
MLFS^b^	NA	NA	<5	ND	
PR	>0.5	>50	≥50% reduction in blasts compared to baseline		
SD	Absence of CR, CR_i_, MLFS, PR, and criteria for PD and LOR not met				
PD^c^			>50% increase in blasts in peripheral blood and/or bone marrow over baseline without first achieving a CR, CR_i_, MLFS, or PR		
LOR	NA	NA	Blasts ≥5% post CR, CR_i_, or MLFS^d^	Reappearance of blasts post CR, CR_i_, or MLFS^d^	
NE	Tissue not available and/or evaluable				

^a^For CR_i_, persistent neutropenia OR thrombocytopenia is permitted.

^b^For MLFS, no hematologic recovery is required. Marrow should not be “aplastic.” At least 200 cells should be enumerated, or cellularity should be at least 10%.

^c^For PD, a minimum 15%-point increase is required in cases with <30% blasts at baseline.

^d^Re-appearance of blasts should be not attributable to any other cause (e.g., regenerating marrow, sepsis, or surgery).

*ANC* absolute neutrophil count, *CR*_*i*_, complete response with incomplete count recovery, *LOR* loss of response, *MLFS* morphologic leukemia-free state, *NA* not applicable, *ND* not detected, *NE* not evaluable, *PD* progressive disease, *PR* partial response.

### Extramedullary disease

The response criteria pertaining to EMD (Table 4) are based on modified Lugano criteria [24]. The presence of splenic and/or liver enlargement was not considered EMD, but instead was evaluated under the CP disease response criteria. Consequently, components of the Lugano criteria pertaining to organ enlargement were not included. However, the presence of discrete splenic and/or hepatic lesions was considered EMD (extralymphatic lesions). Similarly, responses in the BM are addressed by the CP- and BP-specific criteria.

**Table 4:** Response criteria for EMD.

Response and Site	PET/CT-Based Response	CT-Based Response
**Complete response**		
Lymph nodes and extralymphatic sites (including discreet liver and spleen lesions)	Score 1, 2, or 3 on 5-point Deauville scale, with or without a residual mass	Target nodes/nodal masses must regress to ≤1.5 cm in longest transverse diameter No extralymphatic sites of disease
Nonmeasured lesions	Not applicable	Absent
New lesions	None	None
**Partial response**		
Lymph nodes and extralymphatic sites (including discreet liver and spleen lesions)	Score 4 or 5 on 5-point Deauville scale with reduced uptake compared with baseline and residual mass(es) of any size	≥50% decrease in SPD of up to six target measurable nodes and extranodal sites
Nonmeasured lesions	Not applicable	Absent or regressed but no increase
New lesions	None	None
**No response or stable disease**		
Target nodes/nodal masses, extralymphatic sites (including discreet liver and spleen lesions)	Score 4 or 5 with no significant change in FDG uptake from baseline	<50% decrease from baseline in SPD of up to six dominant, measurable nodes and extranodal sites
Nonmeasured lesions	Not applicable	No increase
New lesions	None	None
**Progressive disease**		
At least one target node, nodal mass, or extralymphatic site (including discreet liver and spleen lesions)	Score 4 or 5 with an increase in intensity of uptake from baseline	Progression based on the cross-product of the longest transverse diameter and perpendicular diameter of at least one target node, nodal mass, or extranodal lesion
New lesions	New hypermetabolic lesion consistent with malignancy	Re-growth of previously resolved lesion or new lesion
**Not evaluable**	Imaging not available and/or evaluable	

PET 5-point Deauville scale: 1, no uptake above background; 2, uptake ≤ mediastinum; 3, uptake > mediastinum but ≤ liver; 4, uptake moderately > liver; 5, uptake markedly higher than liver and/or new lesions.

*CT*, computed tomography; *EMD*, extramedullary disease; *FDG*, fluorodeoxyglucose; *PET*, position emission tomography; *SPD*, sum of the products of diameters.

### Overall clinical response based on phase of the disease and involved compartment(s)

The CRC developed composite response criteria for overall clinical response for (1) CP disease in the BM/PB with or without presence of EMD (Table 5); (2) BP disease in the BM/PB with or without the presence of EMD (Table 6); or (3) EMD only since this also represents BP disease (Table 6). For CP disease in the BM/PB (Table 5), six overall response categories are noted: CR, CR_h_, PR, stable disease, loss of response, and progressive disease. For BP disease, seven overall response categories are possible: CR, complete response with incomplete hematologic recovery, morphologic leukemia-free state, PR, stable disease, loss of response, and progressive disease. In both CP and BP diseases, a guiding principle is that overall clinical response is anchored to the lowest quality response among the BM/PB and the EMD disease components.

**Table 5:** Overall clinical responses in chronic-phase disease with or without EMD.

	Chronic Phase Without EMD	Chronic Phase With EMD		
Overall Response	BM/PB Response	BM/PB Response		EMD Response
**CR**	CR	CR	with	CR
**CR**_**h**_	CR_h_	CR_h_	with	CR
**PR**	PR	PR	with	CR or PR
		CR, CR_h_, or PR	with	PR
**SD**	SD	SD	with	CR, PR, or SD
		CR, CR_h_, PR, or SD	with	SD
**LOR**	LOR	LOR	with	CR, PR, SD, or LOR
		CR, CR_h_, PR, SD, or LOR	with	LOR
**PD**	PD	PD	with	CR, PR, SD, LOR, or PD
		CR, CR_h_, PR, SD, LOR, PD	with	PD
**NE**	NE	NE	or	NE

*BM* bone marrow; *CR* complete response, *CR*_*h*_ complete response with incomplete hematologic recovery, *EMD* extramedullary disease, *LOR* loss of response, *NE* not evaluable, *PB* peripheral blood, *PD* progressive disease, *PR* partial response, *SD* stable disease.

**Table 6:** Overall clinical responses in blast-phase disease with or without EMD, or EMD only.

	EMD Only	Blast Phase Without EMD	Blast Phase With EMD		
Overall Response	EMD Response	BM/PB Response	BM/PB Response		EMD Response
**CR**	CR	CR	CR	with	CR
**CR**_**i**_	NA	CR_i_	CR_i_	with	CR
**MLFS**	NA	MLFS	MLFS	with	CR
**PR**	PR	PR	PR	with	CR or PR
			CR, CR_i_, MLFS, or PR	with	PR
**SD**	SD	SD	SD	with	CR, PR, or SD
			CR, CRi, MLFS, PR, or SD	with	SD
**LOR**	LOR	LOR	LOR	with	CR, PR, SD, or LOR
			CR, CR_i_, MLFS, PR, SD, or LOR	with	LOR
**PD**	PD	PD	PD	with	CR, PR. SD, LOR, or PD
			CR, CRi, MLFS, PR, SD, LOR, or PD	with	PD
**NE**	NE	NE	NE	or	NE

*BM* bone marrow, *CR* complete response, *CRi* complete response with incomplete hematologic recovery, *EMD* extramedullary disease, *LOR* loss of response, *MLFS* morphologic leukemia-free state, *NE* not evaluable, *PB* peripheral blood, *PD* progressive disease, *PR* partial response, *SD* stable disease.

## Cytogenetic response (by cytogenetics and fish) and molecular responses

Cytogenetic responses were assessed separately from clinical responses. The criteria for cytogenetic response were developed based on the cytogenetic response criteria proposed for MDS/MPN and are summarized in Table 7 [23]. Molecular responses were defined based on the detection of *FGFR1* fusion transcripts using either semiquantitative or quantitative RT-PCR assays (Table 7). In contrast to the use of international scale, which harmonizes quantitative RT-PCR evaluation of *BCR::ABL1*, no such standardization for molecular monitoring of *FGFR1* and other fusion genes currently exists, but is a high priority for future development. For patients who were enrolled in the FIGHT-203 study with persistent *FGFR1* rearrangement but no morphologic evidence of disease (due to receiving prior therapy), only cytogenetic and/or molecular responses were evaluated.

**Table 7:** Response criteria for cytogenetic and molecular responses.

**Cytogenetic Response**	
Complete cytogenetic response	0% of metaphases with an 8p11 translocation as seen on classic karyotyping with a minimum of 20 metaphases **or** 0% of cells with an *FGFR1* rearrangement (or not exceeding the lower level of detection of the probe) on FISH analysis (break-apart probe) with a minimum of 200 cells evaluated
Partial cytogenetic response	Compared with baseline, ≥50% decrease in metaphases with an 8p11 translocation as seen on classic karyotyping of a minimum of 20 metaphases **or** compared with baseline, decrease of ≥50% of cells with an *FGFR1* rearrangement on FISH analysis (break-apart probe) with a minimum of 200 cells evaluated
Not evaluable	Tissue not available and/or evaluable
**Molecular Response**	
Complete molecular response	No detection of *FGFR1* fusion transcript by RT-PCR (semiquantitative or quantitative RT-PCR required) in target tissue compared with detection of fusion transcript in the same target tissue at baseline
Partial molecular response	≥50% reduction in *FGFR1* fusion transcript by RT-PCR (semiquantitative or quantitative RT-PCR required) in target tissue compared with detection of transcript in the same target tissue at baseline
Not evaluable	Tissue not available and/or evaluable

*FISH* fluorescence in situ hybridization, *RT-PCR* reverse transcriptase-polymerase chain reaction.

Evidence of clonal evolution, e.g., new cytogenetic or molecular genetic abnormalities, should be recorded.

## Concluding remarks

Heterogeneous clinical presentations are observed in patients with MLNs with *FGFR1* rearrangement and other tyrosine kinase fusion genes. This clinical variability presents a challenge for diagnosis and assessment of response. The FIGHT-203 study is the first prospective trial of targeted therapy in MLN with *FGFR1* rearrangement and provided a unique opportunity to generate response criteria, which could adequately address the variable presentations of these diseases. This phenotypic diversity reflects differences in disease acuity (CP vs BP disease), lineage (myeloid vs lymphoid vs mixed phenotype disease), and the variable presence of EMD. We found that these response criteria permit adjudication of the manifold presentations of MLNs, including CP and BP disease with or without EMD, or EMD only. Although these criteria were generated in the context of the FIGHT-203 study of pemigatinib for MLN with *FGFR1* rearrangement, they can also be used to assess therapies for other MLNs with tyrosine kinase fusion genes, including *PDGFRA*, *PDGFRB*, *JAK2*, *FLT3*, and *ETV6::ABL1*. In addition, the response criteria can be applied outside of trials because they incorporate commonly used histopathologic, cytogenetic/FISH, and imaging techniques.

The use of FISH testing is a key adjunct in the diagnosis and follow-up of these disorders, especially when banded metaphases cannot be obtained or are inadequate in number, but its use in response assessment is affected by the different normal cutoffs for different probes and lack of standardized definition of “cytogenetic FISH” response. Therefore, future studies are needed to confirm if there is a difference in FISH results between BM and PB samples and to confirm the correlation between karyotype and FISH results. Similarly, molecular analysis of *FGFR1* fusion transcripts by RT-PCR using a semiquantitative or quantitative assay has not been standardized and is currently not widely available.

These newly proposed response criteria require evaluation in future prospective clinical trials, including whether the categories of response within CP and BP disease correlate with long-term endpoints such as progression-free survival and overall survival.

## Data Availability

Data sharing not applicable to this article as no datasets were generated or analyzed during the current study.

## References

[1] Myeloid/lymphoid neoplasms with eosinophilia and gene rearrangement. In: Swerdlow SH, Campo E, Harris NL, Jaffe ES, Pileri SA, Stein H, et al., (eds). WHO Classification of Tumours of Haematopoietic and Lymphoid Tissues, Revised 4th Edition, 2. Lyon, France: International Agency for Research on Cancer; 2017.

[2] Khoury JD, Solary E, Abla O, Akkari Y, Alaggio R, Apperley JF (2022). The 5th edition of the World Health Organization Classification of Haematolymphoid Tumours: Myeloid and histiocytic/dendritic neoplasms. Leukemia.

[3] Arber DA, Orazi A, Hasserjian RP, Borowitz MJ, Calvo KR, Kvasnicka HM (2022). International Consensus Classification of Myeloid Neoplasms and Acute Leukemias: integrating morphologic, clinical, and genomic data. Blood.

[4] Pozdnyakova O, Orazi A, Kelemen K, King R, Reichard KK, Craig FE (2021). Myeloid/lymphoid neoplasms associated with eosinophilia and rearrangements of *PDGFRA*, *PDGFRB*, or *FGFR1* or with *PCM1-JAK2*. Am J Clin Pathol.

[5] Gerds AT, Gotlib J, Bose P, Deininger MW, Dunbar A, Elshoury A (2020). Myeloid/Lymphoid neoplasms with Eosinophilia and TK fusion genes, Version 3.2021, NCCN Clinical Practice Guidelines in Oncology. J Natl Compr Cancer Netw.

[6] Reiter A, Gotlib J (2017). Myeloid neoplasms with eosinophilia. Blood.

[7] Umino K, Fujiwara SI, Ikeda T, Toda Y, Ito S, Mashima K (2018). Clinical outcomes of myeloid/lymphoid neoplasms with fibroblast growth factor receptor-1 (*FGFR1*) rearrangement. Hematology.

[8] Strati P, Tang G, Duose DY, Mallampati S, Luthra R, Patel KP (2018). Myeloid/lymphoid neoplasms with *FGFR1* rearrangement. Leuk Lymphoma.

[9] Demiroglu A, Steer EJ, Heath C, Taylor K, Bentley M, Allen SL (2001). The t(8;22) in chronic myeloid leukemia fuses BCR to FGFR1: transforming activity and specific inhibition of FGFR1 fusion proteins. Blood.

[10] Reiter A, Cross NC, Gotlib J (2021). Myeloid/lymphoid neoplasms with eosinophilia and TKI fusion genes: treatment. Clin Lymphoma Myeloma Leuk.

[11] Shomali W, Gotlib J (2022). World Health Organization–defined eosinophilic disorders: 2022 update on diagnosis, risk stratification, and management. Am J Hematol.

[12] Metzgeroth G, Schwaab J, Naumann N, Jawhar M, Haferlach T, Fabarius A (2020). Treatment-free remission in *FIP1L1-PDGFRA*–positive myeloid/lymphoid neoplasms with eosinophilia after imatinib discontinuation. Blood Adv.

[13] PEMAZYRE™ (pemigatinib) tablets, for oral use [prescribing information]. Incyte Corporation, Wilmington, DE, 2022.

[14] Goradia A, Bayerl M, Cornfield D (2008). The 8p11 myeloproliferative syndrome: review of literature and an illustrative case report. Int J Clin Exp Pathol.

[15] Kasbekar M, Nardi V, Dal Cin P, Brunner AM, Burke M, Chen YB (2020). Targeted FGFR inhibition results in a durable remission in an FGFR1-driven myeloid neoplasm with eosinophilia. Blood Adv.

[16] Wang T, Wang Z, Zhang L, Wen L, Cai W, Yang X (2020). Identification of a novel *TFG-FGFR1* fusion gene in an acute myeloid leukaemia patient with t(3;8)(q12;p11). Br J Haematol.

[17] Wang Y, Wu X, Deng J, Yu H, Xu R, Zhu Z (2016). Diagnostic application of next-generation sequencing in *ZMYM2-FGFR1* 8p11 myeloproliferative syndrome: a case report. Cancer Biol Ther.

[18] Kreil S, Adès L, Bommer M, Stegelmann F, Ethell ME, Lubking A (2015). Limited efficacy of ponatinib in myeloproliferative neoplasms associated with FGFR1 fusion genes [abstract]. Blood.

[19] Chen J, Deangelo DJ, Kutok JL, Williams IR, Lee BH, Wadleigh M (2004). PKC412 inhibits the zinc finger 198-fibroblast growth factor receptor 1 fusion tyrosine kinase and is active in treatment of stem cell myeloproliferative disorder. Proc Natl Acad Sci USA.

[20] Gotlib J, Kiladjian J-J, Vannucchi A, Rambaldi A, Reiter A, Shomali W (2021). A phase 2 study of pemigatinib (FIGHT-203; INCB054828) in patients with myeloid/lymphoid neoplasms (MLNs) with fibroblast growth factor receptor 1 (FGFR1) rearrangement (MLN^FGFR1^) [abstract]. Blood.

[21] Liu PCC, Koblish H, Wu L, Bowman K, Diamond S, DiMatteo D (2020). INCB054828 (pemigatinib), a potent and selective inhibitor of fibroblast growth factor receptors 1, 2, and 3, displays activity against genetically defined tumor models. PLoS One.

[22] Subbiah V, Iannotti NO, Gutierrez M, Smith DC, Feliz L, Lihou CF (2022). FIGHT-101, a first-in-human study of potent and selective FGFR 1-3 inhibitor pemigatinib in pan-cancer patients with *FGF/FGFR* alterations and advanced malignancies. Ann Oncol.

[23] Savona MR, Malcovati L, Komrokji R, Tiu RV, Mughal TI, Orazi A (2015). An international consortium proposal of uniform response criteria for myelodysplastic/myeloproliferative neoplasms (MDS/MPN) in adults. Blood.

[24] Cheson BD, Fisher RI, Barrington SF, Cavalli F, Schwartz LH, Zucca E (2014). Recommendations for initial evaluation, staging, and response assessment of Hodgkin and non-Hodgkin lymphoma: the Lugano classification. J Clin Oncol.

[25] Döhner H, Estey E, Grimwade D, Amadori S, Appelbaum FR, Büchner T (2017). Diagnosis and management of AML in adults: 2017 ELN recommendations from an international expert panel. Blood.

[26] Shallis RM, Pollyea DA, Zeidan AM (2021). The complete story of less than complete responses: the evolution and application of acute myeloid leukemia clinical responses. Blood Rev.

[27] Shomali W, Gotlib J (2021). Response criteria in advanced systemic mastocytosis: evolution in the era of KIT inhibitors. Int J Mol Sci.

[28] Foucar K, Reichard K, Czuchlewski D Bone Marrow Pathology. Chicago, IL: American Society for Clinical Pathology Press; 2019.

[29] Thiele J, Kvasnicka HM, Facchetti F, Franco V, van der Walt J, Orazi A (2005). European consensus on grading bone marrow fibrosis and assessment of cellularity. Haematologica.

